
JOURNAL INFORMATION
==============================
NLM Title Abbreviation: Wirtschaftsdienst
Journal ID: Wirtschaftsdienst
NLM Journal ID: 101086612

Wirtschaftsdienst (Hamburg, Germany : 1949)

ISSN: 0043-6275

ARTICLE INFORMATION
==============================
PMCID: PMC7560781
PMID: 33082604
DOI: 10.1007/s10273-020-2735-y
Article ID: 2735
Article version: 1
Subjects: Zeitgespräch

Maschinenbau — Strategische Herausforderungen prae, propter et post Corona

Gernandt Johannes johannes.gernandt@vdma.org

Steinwachs Thomas thomas.steinwachs@vdma.org

Wiechers Ralph ralph.wiechers@vdma.org

grid.438937.2 0000 0001 1089 5379 Verband Deutscher Maschinen- und Anlagenbau e.V. (VDMA), Lyoner Straße 18, 60528 Frankfurt a. M., Deutschland
Electronic publication date: 2020 Sep 25
Print publication date: 2020
Volume: 100
Issue: 9
First page: 666
Last page: 670
Copyright: © Der/die Autor(en) 2020
License: Dieser Artikel wird unter der Creative Commons Namensnennung 4.0 International Lizenz (https://creativecommons.org/licenses/by/4.0/deed.de) veröffentlicht.
Open Access wird durch die ZBW — Leibniz-Informationszentrum Wirtschaft gefördert.
License URL: https://creativecommons.org/licenses/by/4.0/

issue-copyright-statement: © The Author(s) 2020

==============================

Das Coronavirus hat die Weltwirtschaft weiter fest im Griff. Hiervon bleibt der Maschinen- und Anlagenbau nicht unberührt. Im ersten Halbjahr 2020 verfehlte die deutsche Maschinenproduktion ihr Vorjahresniveau um 14 %. Die Maschinenexporte verzeichneten im gleichen Zeitraum mit minus 15 % einen Rückgang in ähnlicher Größenordnung. Besonders kräftig fielen die Rückschläge im April und Mai aus. Ende Mai berichteten 98 % der vom Verband Deutscher Maschinen- und Anlagenbau (VDMA) befragten Mitgliedsunternehmen von teils massiven Beeinträchtigungen ihrer Geschäftstätigkeit. In dem Maße, wie sie die zunächst vor allem angebotsseitigen Störungen im Betriebsablauf durch temporäre Betriebsschließungen, angespannte oder gar unterbrochene Lieferketten sowie Reisebeschränkungen in den Griff bekamen, spielten nachfrageseitige Einbußen eine zunehmend wichtige Rolle. Während das erste Quartal mit einem Auftragsminus von lediglich 2 % noch Erwartungen vom nahenden Ende der Rezession zu bestätigen schienen, stand das zweite Quartal mit Orderrückgängen von 30 % ganz im Bann der COVID-19-Pandemie.

Aktuell mehren sich zwar die Signale, dass sich die Wirtschaft nach ihrem tiefen Einbruch im weiteren Jahresverlauf 2020 – wenngleich mühsam – stabilisiert, hier und da sogar belebt, um 2021 auf einen Erholungspfad einzuschwenken. Jedoch zeigt das Wiederaufflammen von Infektionen mit der Folge lokaler Verschärfungen von Gesundheitsauflagen, dass Unternehmen, Wirtschaft und Politik gut beraten sind, sich mit dem Phänomen instabiler Lieferketten nachhaltig zu beschäftigen. Zudem wird das Wiederhochfahren der Wirtschaft nur in dem Maße gelingen, wie angemessene Antworten auf Prä-Corona-Herausforderungen gefunden werden. Dazu zählen die Bewältigung des Strukturwandels in wichtigen Industriesparten, der forcierte Umbau zu mehr ökologischer Nachhaltigkeit sowie die fortgesetzte Digitalisierung von Geschäftsprozessen. Hierauf soll im Folgenden detaillierter eingegangen werden.

## Single Sourcing wird den Herausforderungen unserer Zeit nicht gerecht

Die Corona-Krise hat alte Schwächen des globalen Wertschöpfungssystems schonungslos offengelegt. Zweifelsfrei geht die internationale Arbeitsteilung nachweislich mit deutlichen Wohlfahrtsgewinnen einher, wenn sich alle Marktteilnehmer auf die Produktion jener Güter konzentrieren, bei denen sie über einen komparativen Kostenvorteil verfügen. Jedoch kann dieses System zu einer Ökonomie der vollständigen regionalen Spezialisierung entgleiten. Im Verein mit steigenden Skalenerträgen bei der Bereitstellung von Gütern können sich Unternehmen schnell in einer Situation wiederfinden, in der sie jedes ihrer Vorleistungsgüter nur aus einem einzigen Ursprungsland, vielleicht sogar nur von einzelnen Lieferanten beziehen, im Grenzfall: beziehen müssen.

Dieses „Single Sourcing" kann unter stabilen Normalbedingungen zwar eine effiziente gleichgewichtige Beschaffungsstrategie sein. In einem volatilen Geschäftsumfeld voller Unsicherheiten und Interdependenzen, einer sogenannten VUCA-Welt[1] (Bennet und Lemoine, 2014), hat diese Strategie jedoch ihre Tücken. Zum einen besteht die, wie wir sehen, durchaus realistische Gefahr, dass Handelsbeziehungen von einem Tag zum anderen durch Protektionismus, Sanktionen oder Ausfuhrverbote eingeschränkt und damit wichtige Zulieferer, Partner und Kunden von der Wertschöpfungskette abgeschnitten werden. Zum anderen ist es jederzeit möglich, dass Handelspartner im In- wie im Ausland direkt von unvorhergesehenen lokalen Ereignissen wie Cyberangriffen, politischer Instabilität, Naturkatastrophen oder – wie jüngst – einer Epidemie getroffen werden und somit ihren Lieferverpflichtungen temporär oder gar dauerhaft nicht nachkommen können. Solche exogenen Schocks sind wahrlich keine neuen Risiken. Sie können aber die Produktion der Industriekunden zum Erliegen bringen, wenn diese nicht auf alternative, vom Schock verschonte Zulieferer ausweichen können. Man spricht dann von einem Mangel an Versorgungssicherheit.

1 VUCA steht für: Volatility, Uncertainty, Complexity, Ambiguity.

## Versorgungssicherheit als wichtiges Element der Daseinsvorsorge

Versorgungssicherheit ist nicht nur eine notwendige Bedingung zur Sicherung von Nachfrage und Angebot von Konsum- und Investitionsgütern. Sie ist auch unverzichtbar für die existenzielle Daseinsvorsorge, also die „Sicherung des allgemeinen und diskriminierungsfreien Zugangs zu existentiellen Gütern und Leistungen einschließlich deren Bereitstellung entsprechend der Bedürfnisse der Bürger" (Gabler, 2020). Darunter fallen insbesondere Lebensmittel, Medizinprodukte und Medikamente. So waren es neben leeren Supermarktregalen nicht zuletzt die vorübergehende Knappheit pharmazeutischer Wirkstoffe, deren Produktion stark in China konzentriert ist, fehlende Atemschutzmasken und Beatmungsgeräte, die in der Corona-Krise prompt in allgemeine Forderungen nach einem „Zurückdrehen der Globalisierung" kulminierten. Von dort war es nicht mehr weit zu politischen Appellen für regulatorische Vorgaben inländischer Wertschöpfungsanteile „kritischer" Bereiche oder der gezielten Subventionierung heimischer Hersteller – Maßnahmen, die in ihrer ökonomischen Lenkungswirkung mit Einfuhrzöllen gleichzusetzen sind.

### Renationalisierung bietet keine Lösung und birgt zusätzliche Gefahren

Solche Forderungen sind nicht nur überstürzt, denn die Lieferketten haben sich nach anfänglichen Irritationen wieder gefangen. Auch sitzen Befürworter einem Trugschluss auf: Durch eine Renationalisierung werden nicht nur wichtige Wohlfahrtsgewinne aufgegeben. Sie gewährt überdies auch keinerlei Sicherheit dafür, dass die geänderte Lieferkette von exogenen Schocks verschont bleibt, die im Inland ebenso möglich sind wie an jedem anderen Standort im Ausland. Hilfreich bei der Beurteilung ist zudem ein Wechsel der Blickrichtung: weg aus der Betrachtung des Kunden mit einem potenziellen Versorgungsrisiko hin zu der Perspektive desjenigen, der aufgrund seiner Spezialisierung und komparativer Vorteile über Alleinstellungsmerkmale verfügt.

Der deutsche Maschinenbau ist dafür ein gutes Beispiel. Auf den Bereich der Maschinenindustrie hat sich Deutschland intensiv spezialisiert. Die Branche ist hierzulande mit gut 1,3 Mio. Erwerbstätigen der größte industrielle Arbeitgeber. Dabei bezieht sie rund ein Viertel ihrer Vorleistungen aus dem Ausland. Gleichzeitig gehört der deutsche Maschinenbau selbst zu den stärksten Anbietern auf dem globalen Markt: Bereits seit 2013 hält Deutschland die Position als drittgrößter Maschinenproduzent der Welt. Im Jahr 2019 belief sich der deutsche Maschinenumsatz auf 296 Mrd. Euro und somit ca. 11 % des gesamten Weltmaschinenumsatzes. Stärker waren nur die USA (13 %) und China (33 %). Gleichzeitig hat der Maschinenbau in Deutschland mit 80 % einen sehr hohen Exportanteil. Somit ist die Branche wie kaum eine andere auf funktionierende und offene internationale Märkte angewiesen. Deren Bestand würde aber durch eine protektionistische Politik, deren Beantwortung durch gleichartige Maßnahmen in den Partnerländern wohl nicht lange auf sich warten ließe, massiv gefährdet.

### Globalisierung nutzen, um Resilienz zu steigern

Die jüngsten Erfahrungen mit weltweit zu beobachtenden, national aber durchaus unterschiedlich ausgeprägten Beschränkungen der Lieferfähigkeit im Zuge der COVID-19-Pandemie hat nicht zuletzt im Maschinenbau gezeigt, dass gerade im planmäßigen Ausbau des globalisierten Wertschöpfungsgefüges ein großes Potenzial zur Verminderung seiner Krisenanfälligkeit liegt. Was es hierzu braucht, ist eine ausgewogene Abkehr vom Single-Sourcing-Prinzip hin zu breiteren, regional diversifizierten Liefernetzwerken. Fällt aufgrund eines lokalen exogenen Schocks ein Knoten dieses Netzwerks im In- oder Ausland aus, muss er durch die Verstärkung eines alternativen Knotens kompensiert werden können. Anstelle von Just-In-Time-Lösungen spielt bei einem solchen Schock die Verfügbarkeit von „Just-In-Case"-Lieferungen eine strategische Rolle. Können angebotsseitige Störungen so vermieden werden, verringert sich auch die Gefahr konsekutiver Nachfrageschocks. Je breiter das Netzwerk aufgestellt ist, desto besser kann es die Kraft eines Schocks absorbieren.

### Die Schwächen von gestern sind die Chancen von morgen

Eine Abkehr vom Single Sourcing hat jedoch ihren Preis. Denn sie erfordert auf Seiten der Unternehmen bessere Marktinformationen, eine größere Zahl von Verträgen und nicht zuletzt den Verzicht auf Sonderkonditionen. Das stellt den mittelständisch geprägten Maschinen- und Anlagenbau in Deutschland vor besondere Herausforderungen. Was die Branche nun mehr denn je benötigt, ist die Rückkehr zu einem verlässlichen, regelgebundenen und freien Marktzugang. Damit die zur Schaffung eines resilienteren Globalisierungsgefüges nötigen Aufwendungen möglichst gering bleiben, ist ein Abbau handelsbeschränkender Eingriffe unabdingbar. Dazu gehören Zölle und Handelssanktionen ebenso wie nichttarifäre Handelshemmnisse bei nationalen Standards, Zulassungsverfahren und anderen bürokratischen Hürden. Insbesondere ist auch der europäische Binnenmarkt noch nicht vollendet, wie der Rückfall ins nationale Klein-Klein zu Beginn der Krise gezeigt hat.

Kommen wir damit zum zweiten Betrachtungsgegenstand, der Bewältigung der vielfältigen Strukturherausforderungen, die nicht auf das Konto von Corona gehen. Der britische Staatsmann Winston Churchill soll einmal gesagt haben: „Verschwende niemals eine Krise." Auch die Corona-Krise wirkt in vielen Bereichen wie ein Beschleuniger des Wandels, dem sich die Industrieunternehmen allgemein und der Maschinenbau im Besonderen stellen müssen: die klimafreundliche, nachhaltige Ausrichtung und Weiterentwicklung des Produkt- und Dienstleistungsportfolios für einen globalen Markt. Viele der zur Bekämpfung der jüngsten Wirtschaftskontraktion aufgelegten Konjunkturprogramme zahlen auf diese und andere potenziellen Zukunftstechnologien ein. Beispielhaft genannt seien die Wasserstoffstrategie der Bundesregierung, die Forcierung der Digitalisierung via Glasfaser und 5G-Technologie als Grundlage für neue Geschäftsmodelle und die Beschleunigung des Wandels zur $CO_2$-freien Mobilität.

### Neue Chancen für den Maschinenbau: Grüne Technologien

Auch wenn auf den ersten Blick die genannten Projekte eher anderen Branchen zugeordnet werden, so kommt dem Maschinenbau eine Schlüsselrolle bei der erfolgreichen Umsetzung zu. Denn er ist ein „Enabler" für viele Herausforderungen unserer Zeit. Das gilt auch für das zentrale

Thema Klima, Umwelt und Ressourceneffizienz. Eine Studie des VDMA in Zusammenarbeit mit der Boston Consulting Group (Lorenz et al., 2020) belegt ganz aktuell die bedeutende Rolle des Maschinenbaus als Technologielieferant und Lösungsgeber. Der Maschinen- und Anlagenbau nimmt durch seine Verflechtungen in allen Sektoren eine Schlüsselrolle bei der Reduzierung des globalen Treibhausgas-Ausstoßes ein. Wenn Maschinenhersteller ihren Industriekunden grüne Technologien anbieten und den Umstieg voranbringen, lassen sich die derzeit jährlich 35 Gigatonnen an Emissionen in der Industrie um bis zu 86 % senken, also um bis zu 30 Gigatonnen. Durch den Umstieg auf grüne Technologien entsteht so ein Marktpotenzial von über 300 Mrd. Euro pro Jahr. Das entspricht 12 % bis 15 % des derzeitigen Gesamtumsatzes aller Maschinenhersteller weltweit und summiert sich bis 2050 auf etwa 10 Billionen Euro.

### Digitale Geschäftsmodelle: Unerlässlich zur Stärkung der Krisenresilienz

Ähnlich verhält es sich mit der digitalen Durchdringung industrieller Wertschöpfungsprozesse. In der aktuellen Corona-Krise offenbart sich, wie durch ein Brennglas betrachtet, bei allen Erfolgen auch ein Mangel an Digitalisierungsstrategien in der Industrie. Der Maschinenbau macht hier leider keine Ausnahme. Mehr als die Hälfte (53 %) der im Juni 2020 befragten Softwarehäuser, die als VDMA-Mitglied über Branchenwissen verfügen, geben an, dass bei ihren Kunden aus dem Maschinenbau die Verschiebung oder gar Streichung von Digitalisierungsprojekten ein großes Problem darstellt. Digitalisierung ist jedoch ein wichtiger Baustein, um Resilienz gegen Krisen aufzubauen. Digitale Plattformen, virtuelle Inbetriebnahmen und Simulationen, datengetriebene prädiktive Wartungslösungen sowie ein generell höherer Automatisierungsgrad helfen dem Maschinenbau aus Deutschland, seine Pole-Position im internationalen Wettbewerb auch in Zukunft zu halten.

### Antrieb im Wandel: Chancen ergreifen

Der Transformationsprozess der Mobilität, durch die Pandemie zwar nicht ausgelöst, aber in Folge des verstärkten Rückgriffs auf das Homeoffice und eines geänderten Reiseverhaltens zumindest temporär forciert, birgt mit dem Bedeutungsverfall des klassischen Antriebsstrangs für den Maschinenbau große Risiken, aber auch Chancen auf zusätzliche Wertschöpfung. Denn der Maschinenbau ist hier ebenfalls ein wettbewerbsentscheidender Technologielieferant, sei es bei der Bereitstellung neuer Elemente des veränderten Antriebsstrangs (z.B. Membranen oder Bipolarplatten im sogenannten Cell Stack), klassischer Komponenten im Bereich der Anlagenperipherie (z.B. Filter, Krümmer und Ventile für die Luftführung und das Wasserstoffsystem) oder bei der anschließenden Integration des Antriebsstrangs. Gemeinsam mit seinen Kunden entwickelt der Maschinenbau die zur Herstellung benötigten Investitionsgüter und ermöglicht die für eine flächendeckende Nutzung notwendige Infrastruktur. 11 Mrd. Euro Umsatz für Brennstoffzellenkomponenten sind allein 2040 im Pkw-Segment in Europa möglich. In der Folge können hier rund 68.000 Arbeitsplätze entstehen (VDMA, FEV, 2020). Zudem ist die Maschinenbauindustrie selbst Anwender modernster Antriebstechnologien mit ihren mobilen Arbeitsmaschinen (VDMA, FEV, 2018). Die Brennstoffzelle wird von 2030 an mit einem signifikanten Anteil auch in Nutzfahrzeugen und mobilen Maschinen vertreten sein.

### Wer nicht wagt, der nicht gewinnt

Um die Chancen, die der durch Corona zum Teil beschleunigte strukturelle Wandel birgt, wahrnehmen zu können, kommt es jedoch nicht allein auf den Maschinenbau an. Im Bereich der grünen Technologie ist der Maschinenbau vom Willen seiner Kunden abhängig, Lösungen mit geringerer Energieintensität nachzufragen, deren Entwicklung aufwändiger ist als der Rückgriff auf konventionelle Technologien und die somit zumindest bei geringeren Stückzahlen in der Anfangszeit teurer sind.

Im Bereich der digitalen Geschäftsmodelle wiederum stehen den neuen technologischen Chancen potenzielle Sicherheitsrisiken, wie Datendiebstahl oder Datenerpressung gegenüber, bei deren Bekämpfung nicht allein Kunden und Hersteller ständig am Ball bleiben müssen. Zum Datenschutz bedarf es ergänzend gesetzlicher Rahmenbedingungen und wirkungsvoller Ahndung von Verstößen. Speziell in der Plattformökonomie tritt zu der Gefahr eines Lock-ins, der zu einer starken Marktmacht einzelner Plattformbetreiber führen kann, das zunehmende Risiko eines Lock-outs hinzu, wenn Unternehmen der Zugang zu einer Schlüsselplattform verwehrt wird.

### Politik als wichtiger Partner der Industrie gefordert – unter marktwirtschaftlichen Vorzeichen

Die Entschlossenheit und die Umsetzungsstärke, mit der die politischen Entscheidungsträger in Deutschland im Frühsommer 2020 auf die Corona-Pandemie reagiert haben, verdienten zweifelsfrei Lob. Zu Beginn der Krise ging es um Schnelligkeit. Die Unternehmen benötigten akut genügend Liquidität, um die direkten und indirekten Folgen der Pandemie bewältigen und die Zeit bis zum Wiederanspringen der Nachfrage überbrücken zu können. Insbesondere die Ausweitung des Kurzarbeitergeldes und die vollständige Übernahme der Sozialversicherungsbeiträge durch die Bundesagentur für Arbeit ist ein effizientes und schnell wirksames Instrument. Steuerstundungen und KfW-Kredite haben in vielen Fällen für zusätzliche Liquidität gesorgt. Das Konjunkturpaket ist also insgesamt gelungen. Wie so oft mischt sich in das Lob ein wenig Kritik, warum wir erst jetzt die Kraft hatten, längst überfällige Entscheidungen zu treffen. Degressive Abschreibung, Verbesserungen in der Forschungsförderung, Verlustrückträge, etc. – brauchen wir immer erst eine Krise, um unsere Wettbewerbsfähigkeit zu stärken?

Umso mehr sollten wir jetzt nicht stehen bleiben und stattdessen mutig weitere Schritte zur dauerhaften Stärkung der Wachstumskräfte gehen. Dabei sind insbesondere zwei Fragestellungen wichtig: Wie bekommen wir es hin, dauerhaft auf Innovation und Investitionen zu setzen? Und wie führen wir die enormen Schulden zurück?

Grundlage für die Handlungsfähigkeit der öffentlichen Hand war und ist die Politik der schwarzen Null. Man darf jetzt nicht nur die Schulden erhöhen, man muss gleichzeitig auch die Chance nutzen „alte Zöpfe" abzuschneiden. Langfristiger Schlüssel zum Erfolg ist die Wettbewerbsfähigkeit. Hieran müssen sich sämtliche Maßnahmen messen lassen, damit die Wirtschaft schnell wieder einen nachhaltigen Wachstumspfad einschlagen und dann auch die Schuldenbremse wieder eingehalten werden kann.

Das Konjunkturpaket der Bundesregierung und auch die Elemente des europäischen Recovery-Plans stellen wichtige Weichen für nachhaltige Zukunftsinvestitionen. Entscheidend für den nachhaltigen Erfolg der Maßnahmen sind drei Kriterien: Technologieoffenheit, branchenübergreifende Ausrichtung und Vermeidung von Wettbewerbsverzerrungen. Das gilt auch und gerade in Krisenzeiten. Denn es besteht bei aller Zustimmung die Gefahr, dass die aktuellen Programme die Balance zwischen Staat und Markt zu Ungunsten unseres marktwirtschaftlichen Grundbekenntnisses verschieben. Exitstrategien des Staates bei seinen Eingriffen müssen von Beginn an mitgedacht werden. Der Lust der Politik, über Gebühr in unternehmerisches Handeln einzugreifen oder sich gar in Unternehmen einzukaufen, muss wirkungsvoll entgegengetreten werden. Die Krise darf jetzt nicht instrumentalisiert werden, um durch die Hintertür die Grundregeln der Marktwirtschaft auszuhebeln. Insbesondere ist davor zu warnen, globale Lieferketten unter den Stichworten „Souveränität" und „Resilienz" zu zerreißen. Bund und Länder müssen zweifelsohne beim Pandemieschutz ihre Hausaufgaben machen und Materialen einlagern. Aber hieraus darf nicht der politische Anspruch erwachsen, den freien Handel einzuschränken oder gar alles im eigenen Land selbst zu produzieren. Das ist wohlfahrtsmindernd und nicht effizient und geht letztlich zu Lasten der internationalen Zusammenarbeit.

Dr. Ralph Wiechers ist Mitglied der Hauptgeschäftsführung und Chefvolkswirt des Verbandes Deutscher Maschinen- und Anlagenbau VDMA Frankfurt.

Dr. Thomas Steinwachs arbeitet in der Abteilung Volkswirtschaft und Statistik des VDMA.

Dr. Johannes Gernandt leitet das VDMA Competence Center Wirtschaftspolitische Grundsatzfragen.


Literatur

Bennet, N. und G. J. Lemoine (2014), Crisis Management: What VUCA Really Means for You, Harvard Business Review, Harvard Business School Publishing, January–February 2014 Issue.
Gabler Wirtschaftslexikon (2020), Definition: Was ist „Daseinsvorsorge?“, Revision vom 11.5.2020, https://wirtschaftslexikon.gabler.de/definition/daseinsvorsorge-28469/version-378857 (24. August 2020).
Lorenz, M., M. Lüers, M. Ludwig, S. Rees, H. Rauen, M. Zelinger und R. Stiller (2020), Grüne Technologien für grünes Geschäft, Gemeinschaftsstudie BCG und VDMA, https://web-assets.bcg.com/cd/51/bf13805d4de4a570010010b3dca4/for-machinery-makers-green-tech-creates-green-business-de.pdf (24. August 2020).
VDMA, FEV Consulting (2018), Antrieb im Wandel: Die Elektrifizierung des Antriebsstrangs von Fahrzeugen und ihre Auswirkung auf den Maschinen- und Anlagenbau und die Zulieferindustrie, https://elektromobilitaet.vdma.org/documents/266699/25083160/Antrieb+im+Wandel+-+Broschuere/1bdef884-8c1c-4681-8ac1-ef51bd12fff0 (24. August).
VDMA, FEV Consulting (2020), Antrieb im Wandel: Auswirkungen der Brennstoffzellentechnologie auf den Maschinen- und Anlagenbau und die Zulieferindustrie, https://www.vdma.org/v2viewer/-/v2article/render/49414976 (24. August 2020).
